# Kidney transcriptome response to salinity adaptation in *Labeo rohita*

**DOI:** 10.3389/fphys.2022.991366

**Published:** 2022-10-13

**Authors:** Vemula Harshini, Nitin Shukla, Ishan Raval, Sujit Kumar, Vivek Shrivastava, Amrutlal K. Patel, Chaitanya G. Joshi

**Affiliations:** ^1^ Gujarat Biotechnology Research Centre, Gandhinagar, Gujarat, India; ^2^ Postgraduate Institute of Fisheries Education and Research, Kamdhenu University, Himmatnagar, Gujarat, India

**Keywords:** *L. rohita*, salinity adaptation, transcriptome, kidney, differential gene expression

## Abstract

The increasing salinization of freshwater resources, owing to global warming, has caused concern to freshwater aquaculturists. In this regard, the present study is aimed at economically important freshwater fish, *L. rohita* (rohu) adapting to varying degrees of salinity concentrations. The RNA-seq analysis of kidney tissue samples of *L. rohita* maintained at 2, 4, 6, and 8 ppt salinity was performed, and differentially expressed genes involved in various pathways were studied. A total of 755, 834, 738, and 716 transcripts were downregulated and 660, 926, 576, and 908 transcripts were up-regulated in 2, 4, 6, and 8 ppt salinity treatment groups, respectively, with reference to the control. Gene ontology enrichment analysis categorized the differentially expressed genes into 69, 154, 92, and 157 numbers of biological processes with the *p* value < 0.05 for 2, 4, 6, and 8 ppt salinity groups, respectively, based on gene functions. The present study found 26 differentially expressed solute carrier family genes involved in ion transportation and glucose transportation which play a significant role in osmoregulation. In addition, the upregulation of inositol-3-phosphate synthase 1A (INO1) enzyme indicated the role of osmolytes in salinity acclimatization of *L. rohita*. Apart from this, the study has also found a significant number of genes involved in the pathways related to salinity adaptation including energy metabolism, calcium ion regulation, immune response, structural reorganization, and apoptosis. The kidney transcriptome analysis elucidates a step forward in understanding the osmoregulatory process in *L. rohita* and their adaptation to salinity changes.

## Introduction

One of the main environmental factors affecting the spatial distribution of aquatic life and a significant selective force for local adaptation is salinity (Lemaire et al., 2002; Thiyagarajan et al., 2003; Aktas and Cavdar, 2012). Fish tolerance to variation in salinity depends on their osmoregulatory mechanism, which plays a key role in the maintenance of body fluid homeostasis for survival and efficiency (Rubio et al., 2005; Hwang and Lee, 2007). The kidney is one of the most important osmoregulatory organs, which counterbalances the diffusive loss of major mono and divalent ions through the gills and skin. In freshwater fishes, the kidney produces large volumes of diluted urine by re-absorption of major ions in proximal, distal, convoluted tubules and collecting ducts (Takvam et al., 2021). In recent times, the impact of climate change has resulted in increased salinity levels in fresh water resources (Chong-Robles et al., 2014; Yang et al., 2016; Haque et al., 2020), causing serious effects on the aquatic ecosystem *via* osmotic and ionic stresses on aquatic life.

*L. rohita* (rohu), a local freshwater fish, is distributed throughout South Asia, Southeast Asia, Sri Lanka, Japan, China, the Philippines, Malaysia, and Nepal. Rohu has significantly higher muscle protein content than other major carps (Shakir et al., 2013), the species commands a good market price, and consumer demand makes it an economically important fish. Previous studies in *L. rohita* have investigated the effects of salinity variations on growth performance, hematological and histological changes, and survival (Baliarsingh et al., 2018; Hoque et al., 2020; Murmu et al., 2020); however, the molecular mechanisms involved in salinity adaptation are not well understood.

Studies on the identification of candidate genes involved in adaptation to salinity changes (Zhang et al., 2017) and transcriptome of osmoregulatory organs in fresh and marine water fishes on salinity variations as environmental stressors have been undertaken. The studies highlighted candidate genes of the kidney involved in several pathways such as oxidative phosphorylation, metabolic process, inflammatory response (Chen et al., 2021), energy metabolism, immune-related pathways, and signaling pathways (Xu et al., 2015). In addition, several differentially expressed solute carrier family (SLC) genes as candidate genes in osmotic regulation under salinity stress (Nguyen et al., 2016; Zhang et al., 2017; Zhao et al., 2021) and the myosin and keratin family genes involved in cytoskeletal reorganization (Nguyen et al., 2016; Zhang et al., 2017) have been reported. These studies indicated that fish have differential regulatory mechanisms toward salinity adaptation. Therefore, the present study was conducted to explore the underlying mechanisms of *L. rohita* during salinity tolerance using RNA-seq technology.

The current study is focused on transcriptomic characterization of the *L. rohita* kidney to gradually increased salinity conditions. The aim was to compare the differential expression patterns of genes among varying salt concentrations and identify the genes that play a key role in salinity tolerance. The findings of this study provide a better understanding of how *L. rohita* regulates the genes and pathways to adapt to elevated salinity concentrations.

## Materials and methods

### Salinity stress experimental design

The experiment was conducted at the Postgraduate Institute of Fisheries Education and Research, Kamdhenu University, Himmatnagar, Gujarat. Healthy *L. rohita* fingerlings (>10 g) were acquired and acclimatized to laboratory conditions in 150 L tanks (15 fingerlings/tank) for 7 days with continuous aeration at 27°C ± 5°C. A constant stocking density of 1 gm/L was maintained throughout the experiment, and the water level was maintained accordingly. Feeding was carried out three times a day at the rate of 5% of the body weight. Unused feed and fecal matter were siphoned out, and 25% of water was replaced daily. Later, the fingerlings were randomly divided into control and salinity treatment groups. The experiment was performed in triplicate.

The control group was maintained at 0 ppt throughout the experiment, while in the treatment group, it was gradually increased (1 ppt/day) to the specified salinity (2, 4, 6, and 8 ppt) and thereafter maintained at the particular salinity for 6 days, and on the sixth day, three fish were randomly sampled from the control and treatment groups. Then, the salinity was increased to the next level, and the process was repeated until the last set of samples was collected at 8 ppt on the 32nd day from the start of the experiment. Survival of the fingerlings was observed in the control and treated groups. During experimentation, three fishes from each replicate were randomly selected in respective time intervals according to the salt concentrations and sampled immediately. The collected kidney tissue samples were stored at −80°C in RNA until further RNA extraction. Samples from the control group were also collected simultaneously during sampling at specific salinity. Among the collected samples, one sample each in the control (K1C2, K1C4, K1C6, and K1C8) and treatment (K1T2, K1T4, K1T6, and K1T8) groups at 2, 4, 6, and 8 ppt were processed for the transcriptomic analysis.

### RNA extraction, library construction, and RNA sequencing

Total RNA isolation was carried out using the RNeasy Plus Mini Kit (QIAGEN, Germany), according to the manufacturer’s protocol, and the purity of the isolated RNA was checked with the QIAxpert instrument (QIAGEN, Germany). The quantity and integrity of the RNA were assessed with the Qubit 4 Fluorometer (Thermo Fisher Scientific, United States) and Agilent 2100 Bioanalyzer system (Agilent technologies, California, United States), respectively. Depletion of rRNA was carried out using Low Input RiboMinus Eukaryote System v2 (Thermo Fisher, Massachusetts, United States). Eight sequencing libraries were generated from K1C2, K1C4, K1C6, K1C8, K1T2, K1T4, K1T6, and K1T8 using the TruSeq Stranded Total RNA Library Prep Kit (Illumina, California, United States), following the manufacturer’s instructions, and index codes were added to attribute the sequences of each sample. After purification of enriched DNA fragments, high-throughput sequencing (Miseq/NovaSeq 6000) was performed using paired-end chemistry. The raw sequences were submitted to NCBI Short Read Archive (SRA; Table 1).

**TABLE 1:** Details of the biosample and short-read archive (SRA) submission of the *L. rohita* kidney transcriptome to the NCBI along with the mapping percentage against the reference genome.

Bioproject accession no.	Sample name	Biosample accession no.	Study	Accession no.	Mapping %
PRJNA853878	K1C2	SAMN29416598	SRP384125	SRR19895145	95.51
	K1C4	SAMN29416599	SRP384125	SRR19895144	87.41
	K1C6	SAMN29416600	SRP384125	SRR19895143	83.91
	K1C8	SAMN29416601	SRP384125	SRR19895142	92.89
	K1T2	SAMN29416602	SRP384125	SRR19895141	95.51
	K1T4	SAMN29416603	SRP384125	SRR19895140	85.92
	K1T6	SAMN29416604	SRP384125	SRR19895139	85.89
	K1T8	SAMN29416605	SRP384125	SRR19895138	90.2

### Assembly and differential gene expression analysis

Quality check of data was performed by FastQC (v0.11.9). After quality control, reads were aligned against the reference genome *L. rohita* (Jayanti) breed (GenBank assembly accession: GCA_004120215.1) with segemehl (v0.2.0-418). The reference genome and gene model annotation files (GCA_004120215.1_ASM412021v1) were downloaded from the NCBI, and an index of the reference genome was generated. The read numbers mapped to each gene were counted by featureCounts (v2.0.1). The gene expression levels of each gene were estimated by reads per kilobase of exon per million mapped reads (RPKM). The count data were simulated using the seqgendiff v1.2.3 R package (Gerard, 2020), the analysis of differentially expressed genes between control and salinity-challenged groups was performed with the DESeq R package (v1.34.0), and the genes with the significant *p*-value (<0.05) applied as the threshold were considered as differentially expressed genes (DEGs).

### Enrichment and network analysis

To assess the biological significance of up- and downregulated genes, GO enrichment was performed using ClueGO in Cytoscape (v3.9.1). ClueGO performs functional enrichment in terms of biological processes or pathways which are visualized and grouped to form a network. This highlights the relationship between the enriched genes and their ontology. The default parameters used for ontology were two-sided hypergeometric tests for both enrichment and depletion. Network specificity was set to detail with a kappa score (0.4). Benjamini–Hochberg methodology was applied for statistical pV correction. The DEGs having log2FC < −0.5 OR log2FC > 0.5 and *p*-value <0.05 were considered for network analysis, and in order to increase the readability of the text, the networks were manually arranged.

## Results

### Survivability of *L. rohita* under different salinity concentrations

The survival rate of the fingerlings was found to be 100% up to 8 ppt salinity concentration. This might be due to the slow and gradual increase of the salinity, which helped the fingerlings to adapt to the increased salinity concentrations.

### Data preprocessing and identification of differentially expressed genes

**FIGURE 1:**
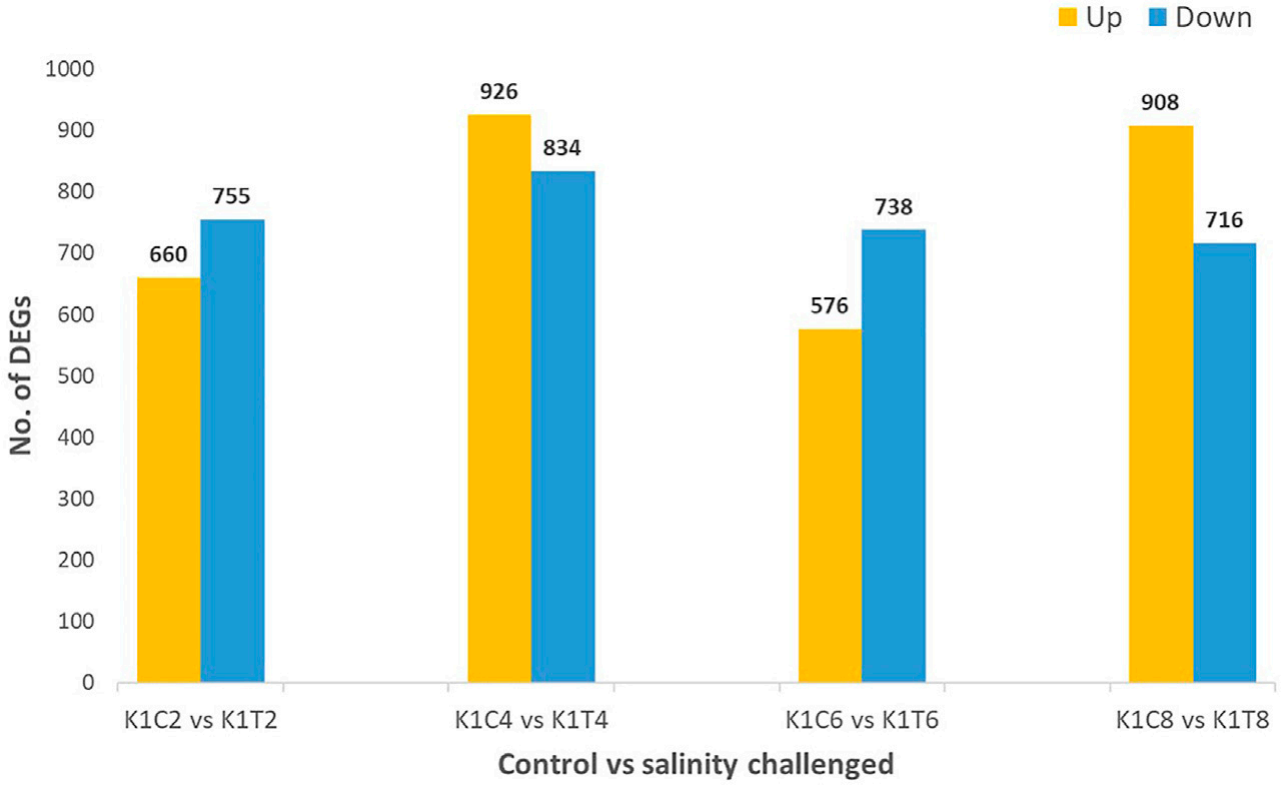
Differentially expressed (up- and downregulated) genes of the *L. rohita* kidney transcriptome at 2, 4, 6, and 8 ppt salinity concentrations (K1C2, K1C4, K1C6, and K1C8; and K1T2, K1T4, K1T6, and K1T8 are the control and salinity treatment groups at 2, 4, 6, and 8 ppt salt concentrations, respectively).

**FIGURE 2:**
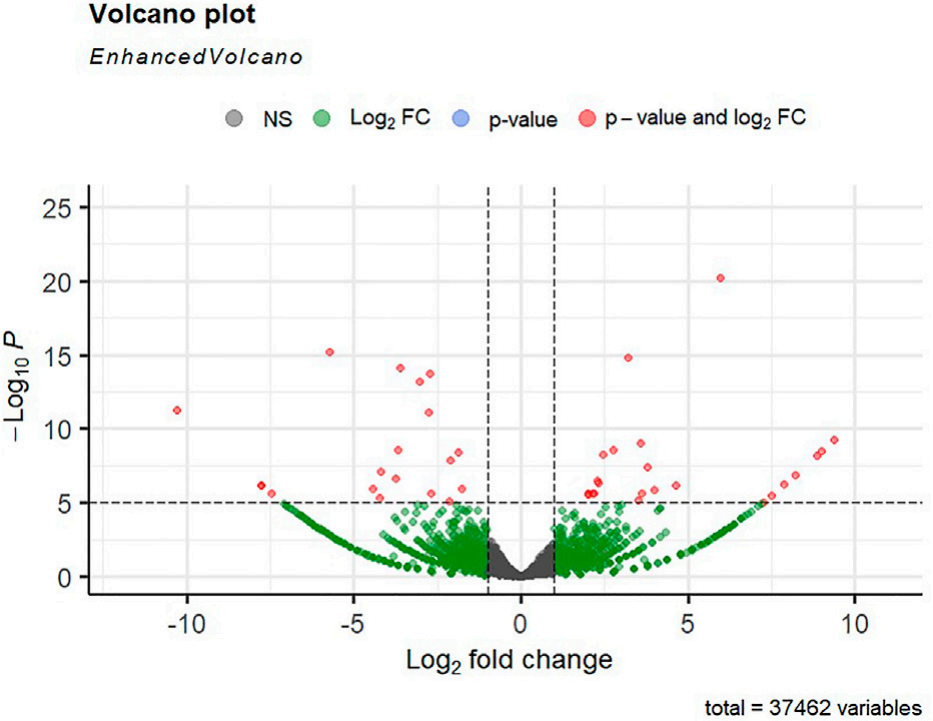
Volcano plot of differentially expressed genes identified between control and 2-ppt salinity group rohu fish. The *X*-axis signifies the Log2 fold change value, and the *Y*-axis indicates the −Log10 *p* value. The ash color dots indicate no significant difference between the two groups. The green dots indicate moderately significant and red dots highly significant differential genes.

**FIGURE 3:**
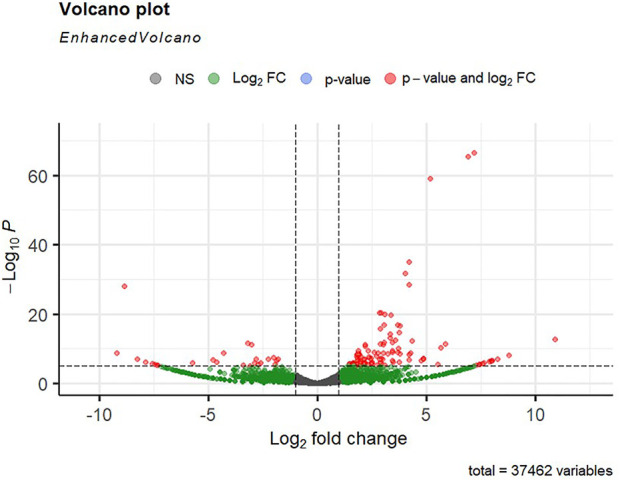
Volcano plot of differentially expressed genes identified between control and the 4-ppt salinity group rohu fish. The *X*-axis signifies the Log2 fold change value, and the *Y*-axis indicates the −Log10 *p* value. The ash color dots indicate no significant difference between the two groups. The green dots indicate moderately significant and red dots highly significant differential genes.

**FIGURE 4:**
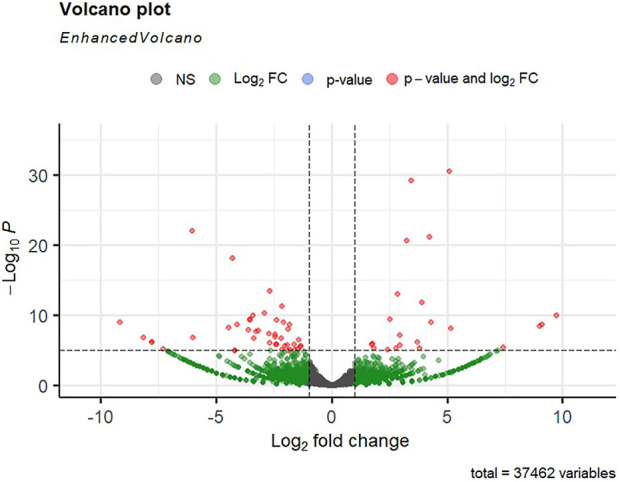
Volcano plot of differentially expressed genes identified between control and the 60-ppt salinity group rohu fish. The *X*-axis signifies the Log2 fold change value, and the *Y*-axis indicates the −Log10 *p* value. The ash color dots indicate no significant difference between the two groups. The green dots indicate moderately significant and red dots highly significant differential genes.

**FIGURE 5:**
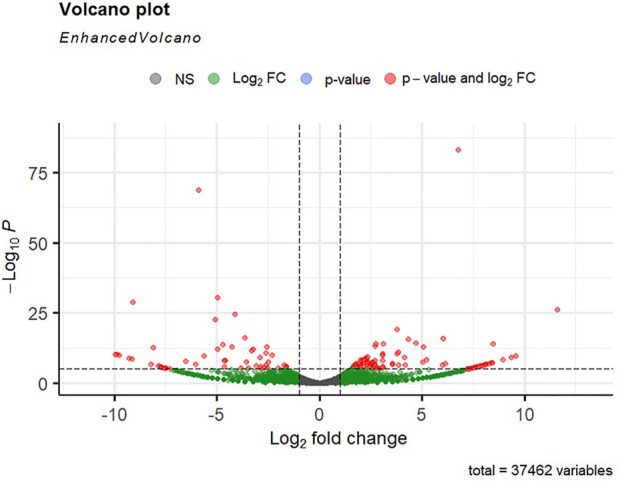
Volcano plot of differentially expressed genes identified between control and the 8-ppt salinity group rohu fish. The *X*-axis signifies the Log2 fold change value, and the *Y*-axis indicates the −Log10 *p* value. The ash color dots indicate no significant difference between the two groups. The green dots indicate moderately significant and red dots highly significant differential genes.

Alignment of reads against the *L. rohita* (Jayanti) reference genome revealed mapping percentages that varied from 83.91% to 95.51% for the control and salinity-challenged groups, respectively (Table 1). A total of 37,462 transcripts were obtained in both the control and treatment groups. The total number of differentially expressed genes (DEGs) (up and down) is presented in Figure 1 with the threshold of a *p*-value < 0.05 and log2 fold change > ± 0.5. The entire list of the DEGs is given in Supplementary Tables S1–S4. The volcano plots representing the differentially expressed genes for 2, 4, 6, and 8 ppt are presented in Figures 2–5, respectively. The hierarchical clustering properly divided the control samples from the salinity-challenged samples representing the differential regulation of genes at 2, 4, 6, and 8 ppt based on normalized counts for differentially expressed mRNA libraries of differentially expressed genes (Supplementary Figures S1–S4).

### Network and gene ontology analysis

**FIGURE 6:**
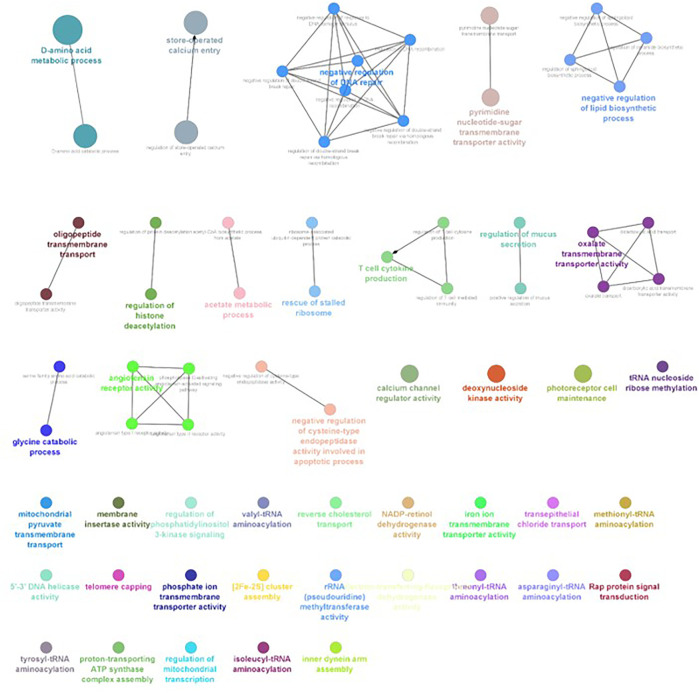
ClueGO enrichment network of the 2-ppt salinity-challenged treatment group DEGs of *L. rohita*, with the *p*-value cut-off <0.05, the interactions of the GO term network determined by functional nodes, and edges shared between differentially expressed genes with a kappa score of 0.4. The generated network has 121 nodes and 164 edges. Each node represents an individual biological process, and the color refers to the GO group. In total, 41 GO groups are present in the network, and edges represent the relationship between the GO terms based on the similarity of their associated genes. The bold font indicates an important GO term that defines the name of the biological process of each group.

**FIGURE 7:**
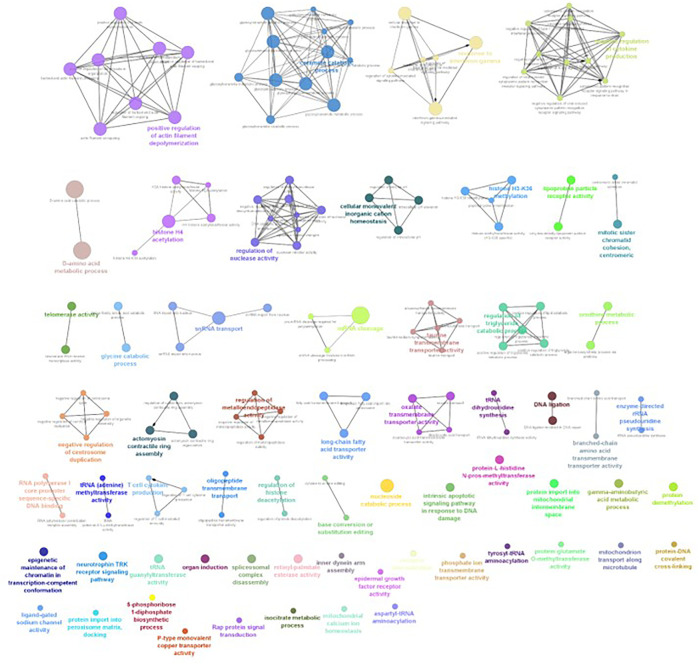
ClueGO enrichment network of the 4-ppt salinity-challenged treatment group DEGs of *L. rohita*, with a *p*-value cut-off <0.05, the interactions of the GO terms network determined by functional nodes, and edges shared between differentially expressed genes with a kappa score of 0.4. The generated network has 227 nodes and 554 edges. Each node represents an individual biological process, and the color refers to the GO group. In total, 60 GO groups are present in the network, and edges represent the relationship between the GO terms based on the similarity of their associated genes. The bold font indicates an important GO term that defines the name of the biological process of each group.

**FIGURE 8:**
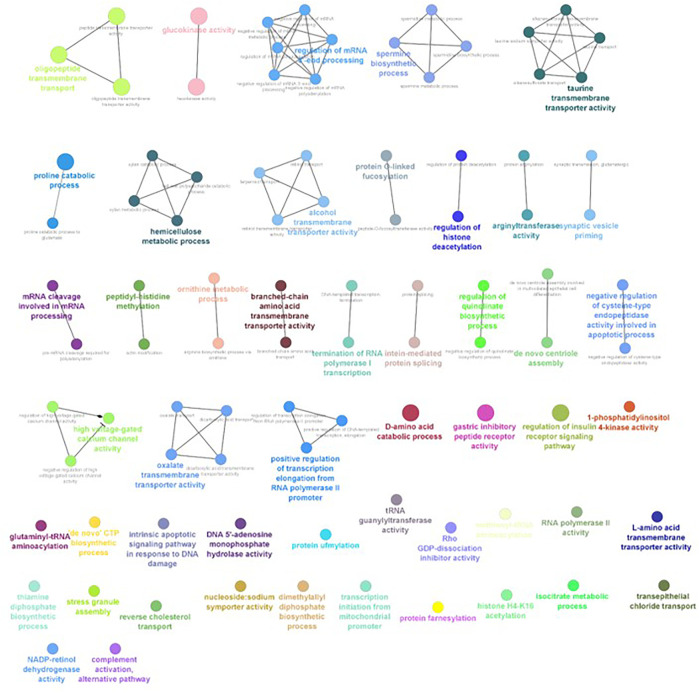
ClueGO enrichment network of the 6-ppt salinity-challenged treatment group DEGs of *L. rohita*, with the *p*-value cut-off <0.05, the interactions of the GO terms network determined by functional nodes, and edges shared between differentially expressed genes with a kappa score of 0.4. The generated network has 148 nodes and 217 edges. Each node represents an individual biological process, and the color refers to the GO group. In total, 49 GO groups are present in the network, and edges represent the relationship between the GO terms based on the similarity of their associated genes. The bold font indicates an important GO term that defines the name of the biological process of each group.

**FIGURE 9:**
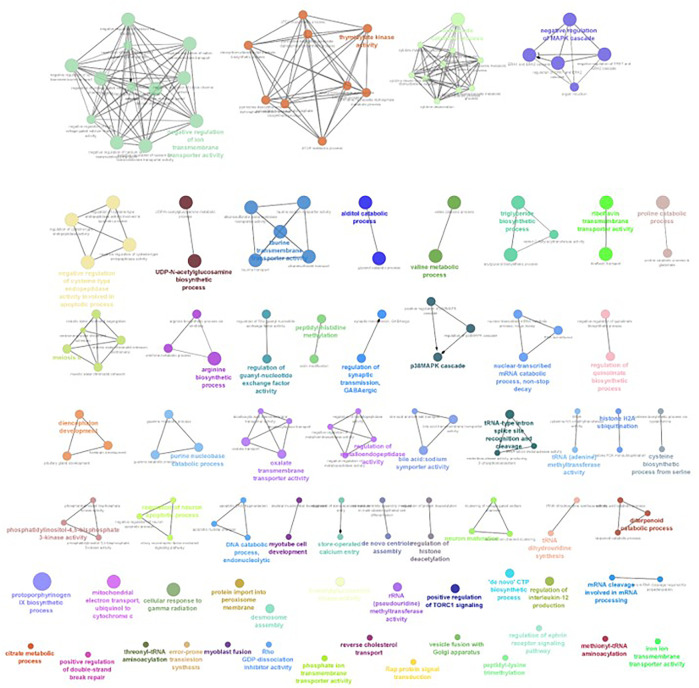
ClueGO enrichment network of the 8-ppt salinity-challenged treatment group DEGs of *L. rohita*, with the *p*-value cut off <0.05, the interactions of the GO terms network determined by functional nodes, and edges shared between differentially expressed genes with a kappa score of 0.4. The generated network has 238 nodes and 550 edges. Each node represents an individual biological process, and the color refers to the GO group. In total, 63 GO groups are present in the network, and edges represent the relationship between the GO terms based on the similarity of their associated genes. The bold font indicates an important GO term that defines the name of the biological process of each group.

The differential gene expression analysis provided information regarding the genes functionally related to the salinity stress in *L. rohita*. However, in the differential expression analysis, each gene is considered independently, while in reality, the expressions of gene and gene products are interconnected and function in networks. Hence, to discover the genes which are co-expressed, GO enrichment analysis was conducted using ClueGO, which has also generated the visualization of interactions between different biological pathways (Figures 6–9) and thus grouped into biological processes based upon the similarity in the functionality of the genes. In 2, 4, 6, and 8 ppt salinity concentrations, the most enriched biological processes are given in Table 2 (Supplementary Figures S5–S8). A complete list of all enriched biological processes along with GO ids and associated genes is reported in Supplementary Tables S5–S8.

**TABLE 2:** List of predominantly enriched GO terms with their biological processes (based on the number of genes involved) of the *L. rohita* kidney transcriptome under increased salinity concentrations obtained from the ClueGO network analysis of DEGs.

Salinity concentration (ppt)	GO ids	Biological process
2	0046416	D-amino acid metabolic process
	0090481	Pyrimidine nucleotide-sugar transmembrane transporter activity
	0005246	Calcium channel regulator activity
	0002115	Store-operated calcium entry
	0045494	Photoreceptor cell maintenance
	0019136	Deoxynucleoside kinase activity
4	0046416	D-amino acid metabolic process
	0046514	Ceramide catabolic process
	0006379	mRNA cleavage
	0030836	Positive regulation of actin filament depolymerization
	0034341	Response to interferon-gamma
	0051030	snRNA transport
	0009164	Nucleoside catabolic process
6	0019478	D-amino acid catabolic process
	0016519	Gastric inhibitory peptide receptor activity
	0046626	Regulation of the insulin receptor signaling pathway
	0035672	Oligopeptide transmembrane transport
	0004340	Glucokinase activity
	0006562	Proline catabolic process
8	0032413	Negative regulation of the ion transmembrane transporter activity
	0009164	Nucleoside catabolic process
	0005368	Taurine transmembrane transporter activity
	0043409	Negative regulation of MAPK cascade
	0043154	Negative regulation of the cysteine-type endopeptidase activity involved in the apoptotic process
	0019432	Triglyceride biosynthetic process
	0006526	Arginine biosynthetic process
	0032217	Riboflavin transmembrane transporter activity
	0019405	Alditol catabolic process
	0071480	Cellular response to gamma radiation
	0006122	Mitochondrial electron transport, ubiquinol to cytochrome c
	0006782	Protoporphyrinogen IX biosynthetic process
	0006048	UDP-N-acetylglucosamine biosynthetic process
	0006573	Valine metabolic process

The overall GO enrichment analysis of 2, 4, 6, and 8 ppt salinity concentrations revealed many specific modified pathways in relation to salinity stress. The biological processes predominantly enriched are involved in osmoregulation (GO:0005246, GO:0015114, GO:0008331, GO:1901841, GO:1901842, GO:0034766, GO:0032413, GO:1904063, GO:2001258, GO:0015114, and GO:0045162), osmolyte production (GO:0014066, GO:0005368, GO:0005369, GO:0046934, GO:0052812, GO:0052813, GO:0004430, and GO:0015734), energy metabolism (GO:0006850, GO:0019427, GO:0006083, GO:1902001, GO:0005324, GO:0050996, GO:0090207, GO:0090208, GO:0010896, and GO:0010898), calcium ion regulation (GO:0005246, GO:0002115, GO:2001256, GO:0008331, GO:1901841, GO:1901842, GO:0051926, GO:1903170, and GO:1901020), immune response (GO:0002369, GO:0002709, GO:0001959, GO:0034341, GO:0060330, GO:0071346, GO:0060333, GO:0032480, and GO:0060334), and apoptosis (GO:0008630, GO:0030262, GO:0006309, and GO:0043523).

The GO enrichment analysis is restricted to *L. rohita*, not a model species, and thus, rohu-specific annotations are not readily recognized. Therefore, we further explored the differentially expressed genes individually and observed genes involved in hypersalinity stress, viz., genes involved in the activation of hypoxia-related pathways, endoplasmic reticulum-associated protein degradation, cell cycle arrest, FOXO signaling pathway, and the genes involved in the structural reorganization and detoxification under environmental shift (migration from freshwater to saltwater).

## Discussion

A significant number of genes were differentially expressed upon salinity adaptation in *L. rohita*. We have discussed key components of categories and their potential functions in response to salinity variations.

Earlier, it has been reported that 20%–62% of the total energy of a fish is spent on osmoregulation under elevated salinity levels (Bœuf and Payan, 2001). In our study, there was upregulation of acyl-CoA synthetase (ACS), polyketide synthase (PKS), and malonyl acyl carrier mitochondrial (MCAT) key enzymes utilized in the energy metabolism. ACS plays an important role in the production of acetyl-CoA, which involves fatty acid synthesis and the tricarboxylic acid (TCA) cycle (Fujino et al., 2001). MCAT is an important metabolic enzyme in the saturated fatty acid synthesis pathway, and its upregulation has a positive effect on the accumulation of C16 and C18 fatty acids (Simon and Slabas, 1998). Polyunsaturated fatty acids (PUFA), produced through the PKS pathway, are catalyzed by polyketide synthase (PKS) (Metz et al., 2001). The upregulated PKS indicated the increased production of PUFA as the salinity tolerance of *L. rohita*, as concluded by Sui et al. (2007) in Chinese mitten crab larvae. Along with the genes involved in the energy metabolism, several genes with functions of transporting molecules were differentially expressed. Corresponding to the overexpression of lipid metabolic enzymes, lipid transporters such as apolipoproteins (*APOL6*, *APOB*, *APOE*, *APOA1*, and *APOL3*) and *SLC22A5* were upregulated. *APOE* and *APOB* act as ligands for the lipoprotein receptors and are involved in the transportation of lipids. Similar results were reported in spotted sea bass under salinity stress-responsive transcriptomic analysis (Zhang et al., 2017). This inferred that the lipid metabolism plays an important role in energy production to maintain osmoregulation under salinity stress in *L. rohita*. The differential expression of several solute carrier family genes that participate in glucose transportation, i.e., *SLC2A2*, *SLC2A1*, *SLC2A9*, *SLC2A11*, and *SLC5A1* and in amino acid transportation, *SLC7A3*, was observed in *L. rohita*. During high salinity conditions, the free amino acids can be metabolized in order to provide the ATP necessary for the metabolic process (Nguyen et al., 2016) and can also be potentially involved in the regulation of intracellular osmotic pressure (Venkatachari, 1974; Tseng and Hwang, 2008).

In addition to this, there was differential expression of other SLC family genes (*SLC13A1*, *SLC41A1*, *SLC26A6*, *SLC4A4*, *SLC12A1*, *SLC12A3*, *SLC12A4*, *SLC20A1*, and *SLC34A1*) which indicated the reduced re-absorption levels of the major ions in the kidney as the fish were adapting to the salt water. This finding coincides with that of Watanabe and Takei (2011) for euryhaline fish, where migration from freshwater to saltwater caused a drastic change in ion regulation in the opposite direction, from absorption to excretion in osmoregulatory organs such as the gills, kidney, and intestines. Interestingly, there was also an enrichment of the calcium channel regulatory activity, regulation of store-operated calcium entry biological processes, and also differential regulation of *phospholipase C beta 4 (PLCB4)* and *inositol 1,4,5 trisphosphate receptor (IP3R)* type II genes, causing the release of calcium from the endoplasmic reticulum (Vervloessem et al., 2014). Calcium has been known to be involved in many biological pathways including the apoptotic process (Jiang et al., 1994) and also cell survival through cAMP-responsive element binding (CREB), a Ca^2+^-activated transcription factor (Bito and Takemoto-Kimura, 2003; Persengiev and Green, 2003). The differential expression of CREB-regulated transcription co-activators and protein kinases (phosphorylation of serine, which is related to CREB activity) (PK, 1992) demonstrates that Ca^2+^ activates the CREB as a pro-survival transcription factor under hyperosmotic environmental conditions.

**FIGURE 10:**
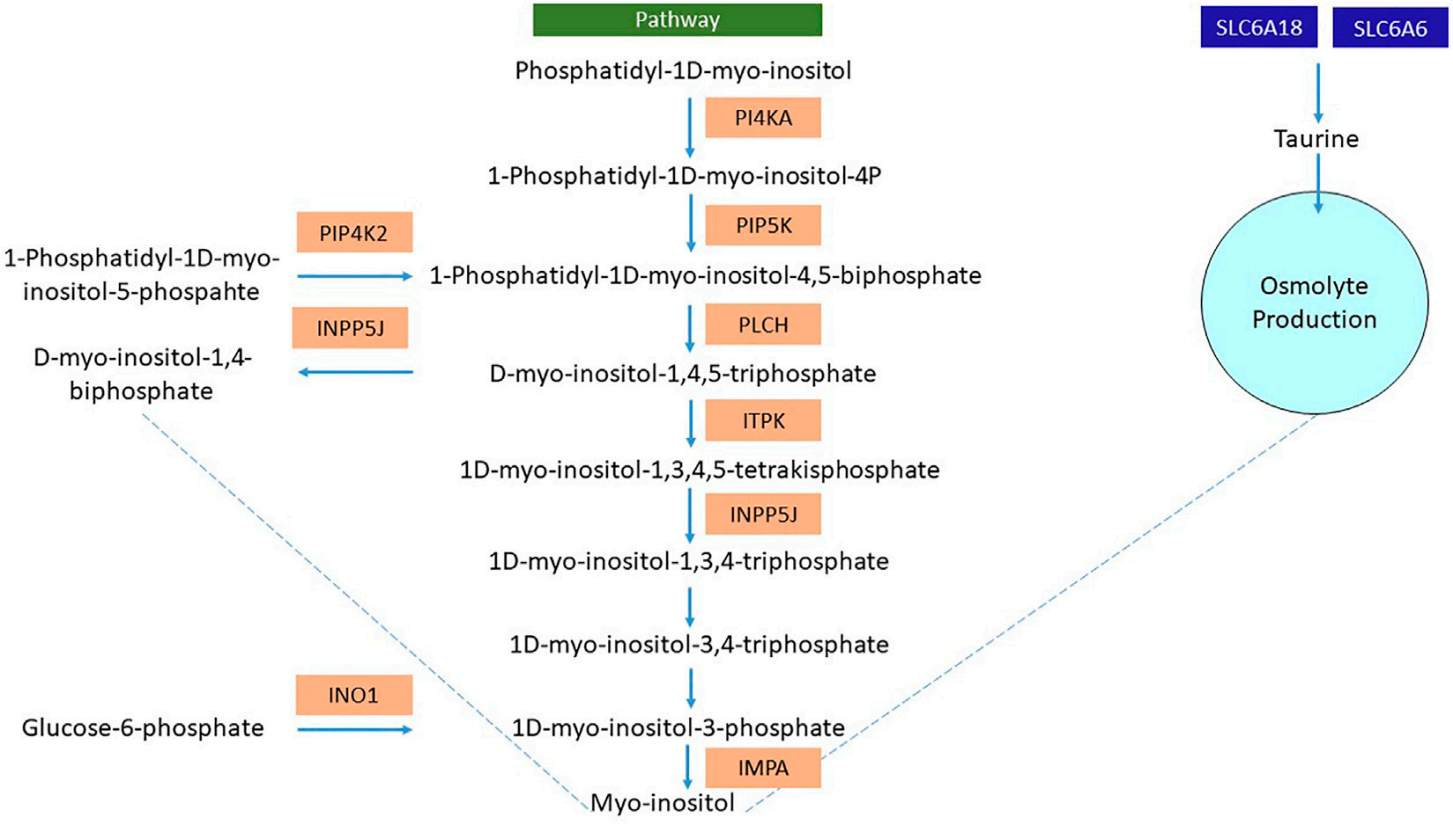
Representative flowchart of differentially expressed genes (highlighted in orange) involved in the osmolyte production (myo-inositol and taurine) of the *L. rohita* kidney after transferring to a high salt concentration. PI4KA, phosphatidylinositol 4-kinase alpha; PIP5K, phosphatidylinositol-4-phosphate 5-kinase; PLCH, phospholipase C-like protein; ITPK, inositol trisphosphate 3-kinase; INPP5J, phosphatidylinositol 4,5-bisphosphate-5-phosphatase; PIP4K2, phosphatidylinositol 5 phosphate-4-kinase; INO1, inositol-3-phosphate synthase; SLC6A6, solute carrier family 6 member 6; SLC6A18, solute carrier family 6 member 18.

Likewise, *L. rohita* also showed various interesting mechanisms involved in osmolyte production to counterbalance the osmotic pressure induced due to hypersalinity. The upregulation of the inositol-3-phosphate synthase 1A (*INO1*) enzyme, *SLC6A6*, and *SLC6A18* was noticed. The *INO1* gene converts glucose-6-phosphate to 1D myo-inositol-3-phosphate, eventually converting to myo-inositol (Sacchi et al., 2013). The enzymatic action of phosphatidylinositol 4-kinase alpha and phosphatidylinositol 5-phosphate-4-kinase through a series of events leads to the myo-inositol synthesis (KEGG pathway id: 00562) (Figure 10). *SLC6A6* and *SLC6A18* genes involved in the sodium and chloride-dependent taurine transport determine the role of taurine as an osmolyte in the acclimatization of *L. rohita* to hypersalinity conditions. Similar conclusions were also made by Gonçalves et al. (2011) in Nile tilapia.

The significant differential expression of the myosin gene family members (*MYO1H*, *MYO6*, *MYO3B*, *MYO5B*, *MYO28A*, *MYH9*, *MYO9A*, *MYO7A*, and *MYLK*), keratin (KRT-8), and collagen family genes in rohu under a high salt concentration was observed. The genes encoding the structural components of the cytoskeleton induced at hypersalinity conditions might be to adjust the elements for cell reorganization during cell volume regulation (Nguyen et al., 2016) under osmotic stress to maintain the equilibrium. The increased expression of myosin light chain genes was also found in *Pangasianodon hypophthalmus* (Nguyen et al., 2016) and *L. maculatus* (Zhang et al., 2017) under salinity stress, whereas the expression of keratin-related genes was observed in catfish under heat stress (Liu et al., 2013). In rohu, under high salt concentration, the gradual upregulation of several cytochrome *P450* genes and several isoforms of glutathione-S-transferase (GST) (Supplementary Tables S1–S4) was observed among the four salinity treatment groups. *CYP1A* is believed to be important in acclimatization of freshwater fishes to a higher salt concentration, and increased expression was also reported in *Oncorhynchus mykiss* (Leguen et al., 2010), *Geophagus mirabilis* (Evans and Somero, 2008), and *Oncorhynchus kisutch* (Lavado et al., 2014) gill transcriptome studies. Glutathione-S-transferase (GST) quenches reactive molecules and catalyzes glutathione conjugation to hydrophobic and electrophilic substrates, thus protecting the cells against an oxidative burst (Kumar and Trivedi, 2018). The results coincided with transcriptomic findings of *Anguilla anguilla* (Kalujnaia et al., 2007) and *P. hypophthalmus* (Nguyen et al., 2016).

Furthermore, the exploration of significantly expressed genes manifested an ER-associated degradation (ERAD) of misfolded proteins (KEGG term ID: 04141) (Supplementary Figure S9) as a consequence of the unfolded protein response. The overexpressed mannosyl-oligosaccharide alpha-1,2-mannosidase (*MAN1A1*) gene accelerated the ER-associated degradation of misfolded proteins (Hosokawa et al., 2007; Ogen-Shtern et al., 2016). The conceptual hypothesis from the differentially expressed genes is that terminally misfolded proteins were recognized by ERAD substrate recognizers, BiP (*HSPA5*), a molecular chaperon, and endoplasmic reticulum lectin 1 (*ERLEC1*) gene (Nakatsukasa and Brodsky, 2008) and translocated to the cytoplasm called retrotranslocation. Then, ERAD substrates interacted with the E3 ubiquitin ligase complex (*DNAJB2*, *HSPA1*, and *UBE2D*) in the cytoplasm, and the ubiquitinated substrates were delivered to the proteasome by the p97 complex (VCP), an ATP-requiring hexameric AAA ATPase (Ye et al., 2001; Jarosch et al., 2002; Rabinovich et al., 2002). There was an upregulation of *HERPUD1* gene, which also acted as a shuttle factor for delivering the ubiquitinated ERAD substrates to the proteasome (Okuda-Shimizu and Hendershot, 2007; Huang et al., 2014). There was also a differential expression of several proteasomal subunits (*PSMD2*, *PSMD6*, *PSMD10*, *PSMD11*, *PSMD12*, *PSMB2*, *PSMB6*, *PSMB7*, *PSMG4*, and *PSME3*) which explains the proteasomal degradation of ubiquitinated proteins through an ATP-dependent mechanism (Motosugi and Murata, 2019). Corresponding to this, there was also the upregulation of NEK5, polyubiquitin B, C, and cathepsins involved in removal or degradation of misfolded proteins (Pratt et al., 2010; Kaminskyy and Zhivotovsky, 2012; Lin and Qin, 2013; Bianchi et al., 2019).

In addition, an elevated salt concentration also had an influence on the immune response in *L. rohita.* There was a differential expression of genes involved in the phagosome pathway (KEGG term id: 04145) (Supplementary Figure S10). Manifestation of the phagocytic process under salinity stress was also reported in *Oreochromis mossambicus* (Jiang et al., 2008) and *P. hypophthalmus* (Schmitz et al., 2017). Maturation of phagosomes leads to the antigen presentation by MHC complex I/II and causes the activation of T cells (Krogsgaard et al., 2005). Moreover, there was an enrichment of biological processes such as the regulation of T-cell-mediated immunity and regulation of T-cell cytokine production. Continuous downregulation of the lysozyme G activity was found across the four salinity-challenged groups, which might be due to the increased complement activity, as also observed in *O. mossambicus* after transferring to saltwater (Jiang et al., 2008).

Transfer of *L. rohita* from freshwater to saltwater stimulated the pathways involved in prolonging the lifespan of the species. Among them, the FOXO signaling pathway (KEGG term ID: 04068) (Supplementary Figure S11) either induced or inhibited apoptosis, depending on the level of stress. In case of prolonged stress, apoptosis of the abnormal cells prolonged the longevity of the organism (Carter and Brunet, 2007). The cellular senescence pathway (KEGG term ID: 04218) (Supplementary Figure S12) caused cell cycle arrest and can be triggered in response to different kinds of stress. Under hypersalinity stress, cellular senescence may be an alarming response to prevent the multiplication of abnormal cells and protect the animal (Kumari and Jat, 2021). There was also enrichment of the apoptotic pathway (KEGG term ID: 04210) (Supplementary Figure S13). Under the salinity effect, these three pathways might balance cell proliferation and survival to maintain cellular homeostasis in *L. rohita*.

## Conclusion

The current experiment investigated the transcriptomic response of the *L. rohita* kidney treated under four different salt concentrations (2, 4, 6, and 8 ppt). From the molecular aspect, during adaptation to high salinity levels, *L. rohita* produced significant changes in osmoregulation, energy metabolism, hypoxia, protein processing, immune response, structural reorganization, and detoxification, suggesting the importance of core components of the kidney in salinity acclimatization. The differential gene expression patterns and their pathways give insights into the molecular mechanisms involved in the salinity adaptation in rohu. Our findings also support the earlier studies on *L. rohita* for the plasticity of salinity tolerance and the maintenance of individuals at low-to-moderate salt concentrations without affecting their performance. The transcriptomic information also suggests the scientific basis of response to climate change, causing increased salinity levels in freshwater resources and is also helpful to scientists involved in research on freshwater fishes.

## Data Availability

The datasets presented in this study can be found in online repositories. The names of the repository/repositories and accession number(s) can be found at: https://www.ncbi.nlm.nih.gov/, SRR19895145; https://www.ncbi.nlm.nih.gov/, SRR19895144; https://www.ncbi.nlm.nih.gov/, SRR19895143; https://www.ncbi.nlm.nih.gov/, SRR19895142; https://www.ncbi.nlm.nih.gov/, SRR19895141; https://www.ncbi.nlm.nih.gov/, SRR19895140; https://www.ncbi.nlm.nih.gov/, SRR19895139; and https://www.ncbi.nlm.nih.gov/, SRR19895138.
