# Supplementary material for: Kidney transcriptome response to salinity adaptation in Labeo rohita
Source: Front Physiol. 2022 Oct 13;13:991366. doi: 10.3389/fphys.2022.991366 (PMC9606766; doi:10.3389/fphys.2022.991366)
Supplement: Supplementary file 1 [file DataSheet1.ZIP › Supplementary figures.docx]

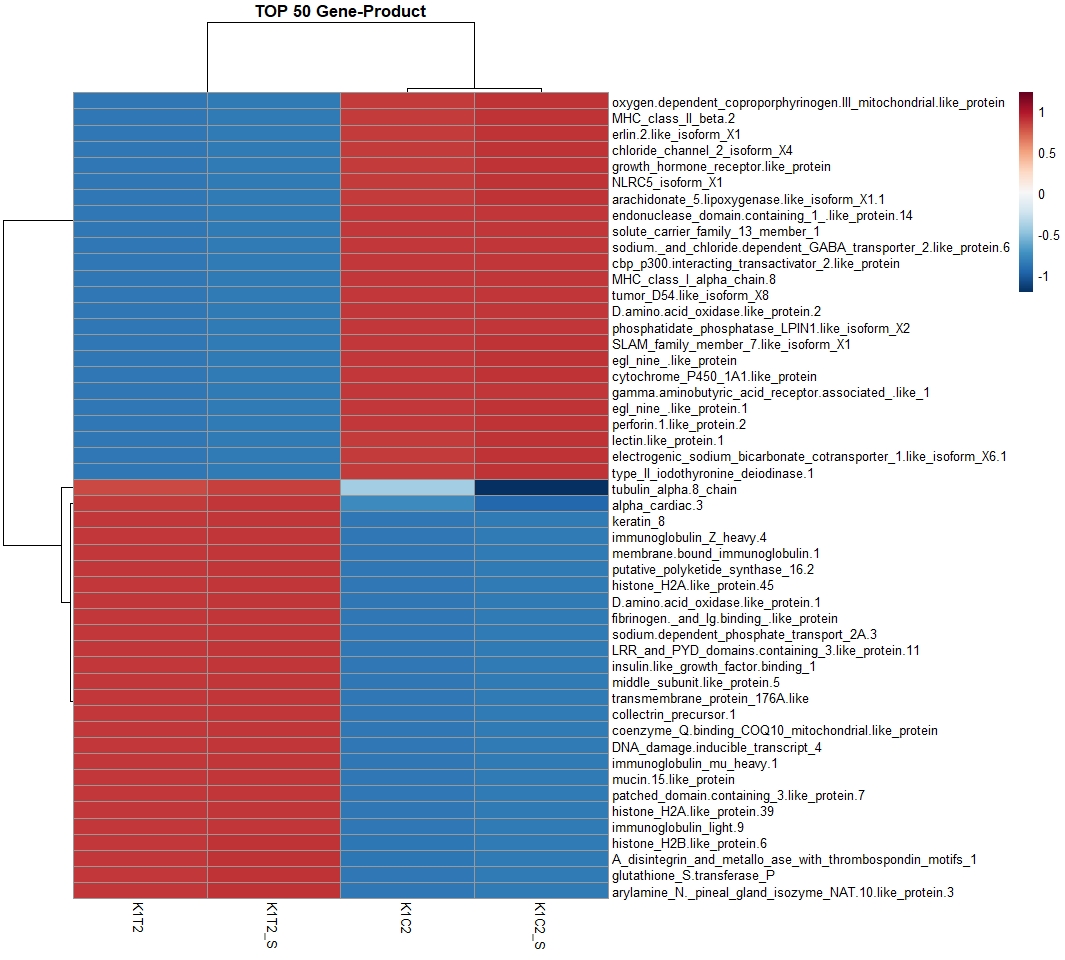


Supplementary figure 1. Heat map of RNA-seq transcriptome of *Labeo rohita* kidney for top 50 significantly differentially expressed genes between control (K1C2 and K1C2-S) and 2 ppt salinity treated samples (K1T2 and K1T2-S). The heat map showing normalized read counts (rows) for transcripts and four samples (columns). The blue and red colour corresponds to the down and up regulated genes in respective samples


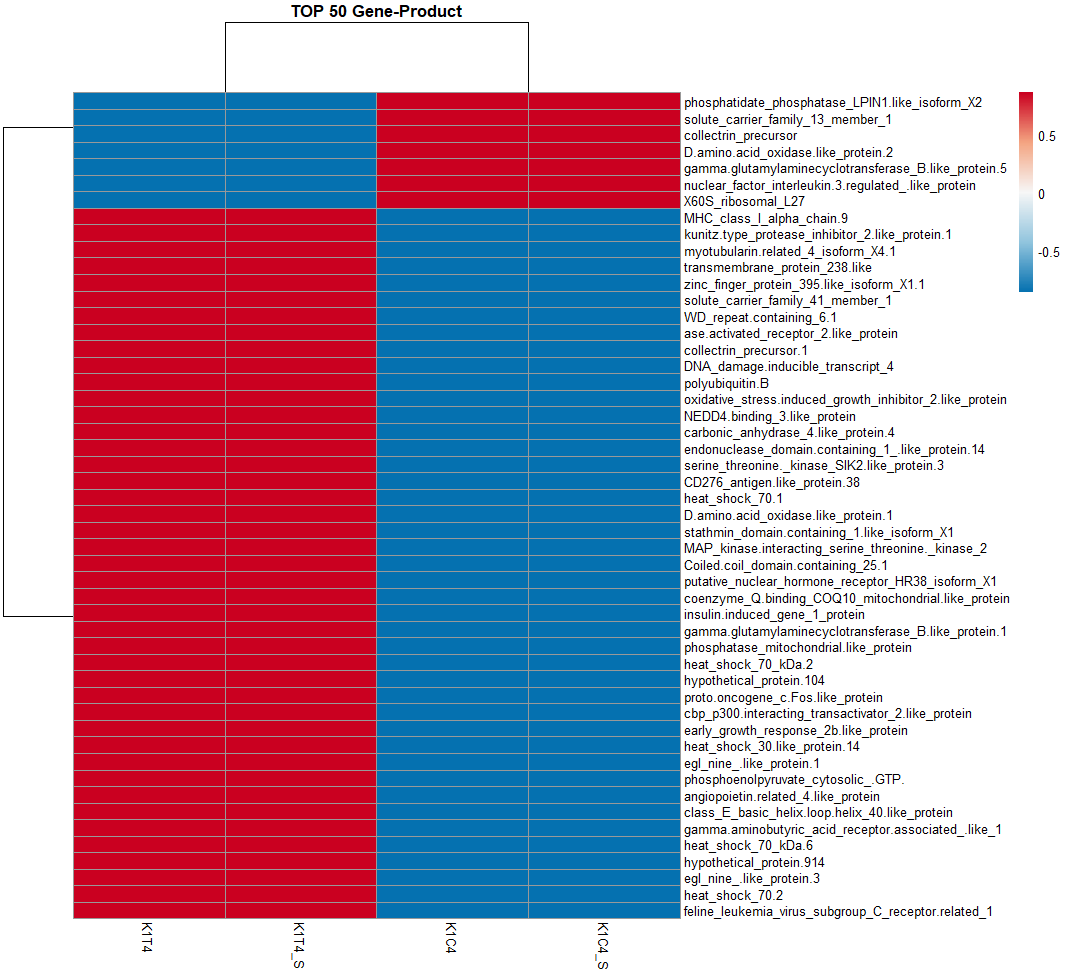
Supplementary figure 2. Heat map of RNA-seq transcriptome of *Labeo rohita* kidney for top 50 significantly differentially expressed genes between control (K1C4 and K1C4-S) and 4 ppt salinity treated samples (K1T4 and K1T4-S). The heat map showing normalized read counts (rows) for transcripts and four samples (columns). The blue and red colour corresponds to the down and up regulated genes in respective samples


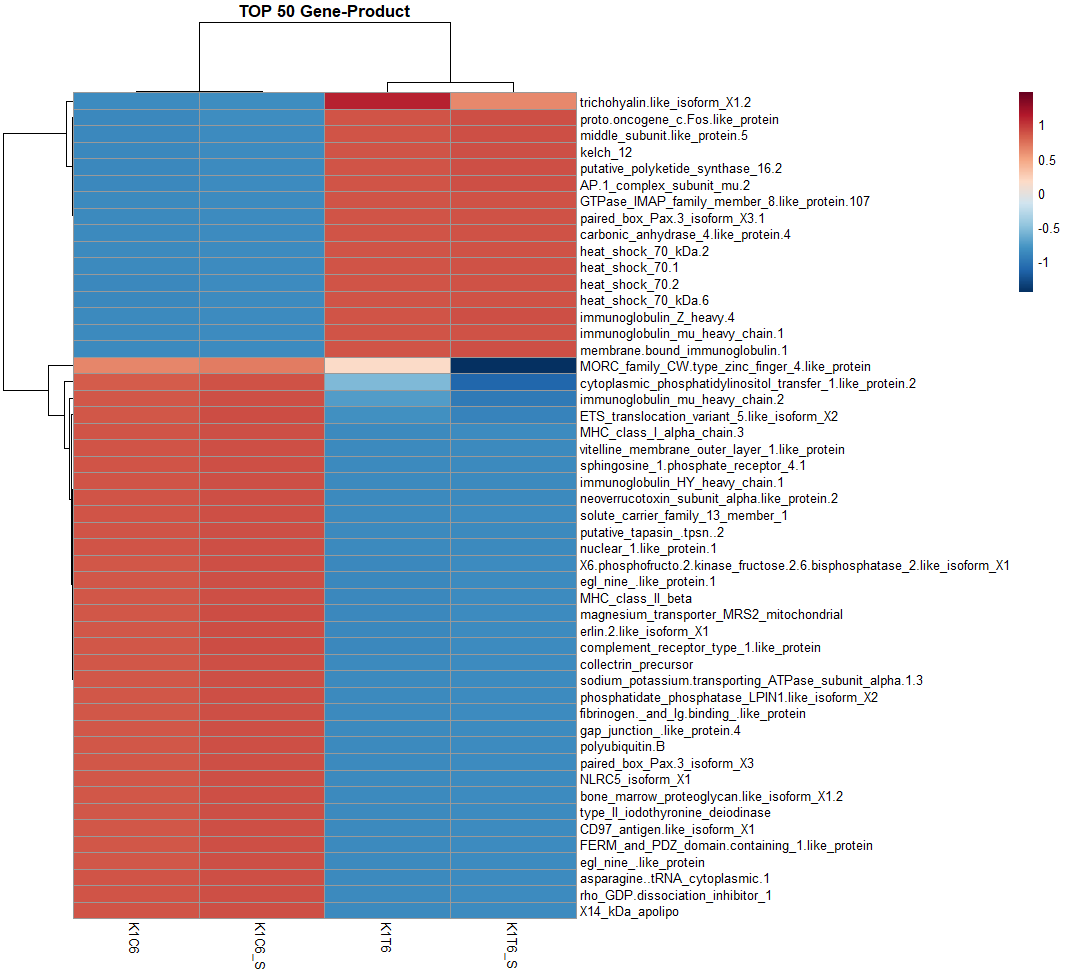
Supplementary figure 3. Heat map of RNA-seq transcriptome of *Labeo rohita* kidney for top 50 significantly differentially expressed genes between control (K1C6 and K1C6-S) and 6 ppt salinity treated samples (K1T6 and K1T6-S). The heat map showing normalized read counts (rows) for transcripts and four samples (columns). The blue and red colour corresponds to the down and up regulated genes in respective samples


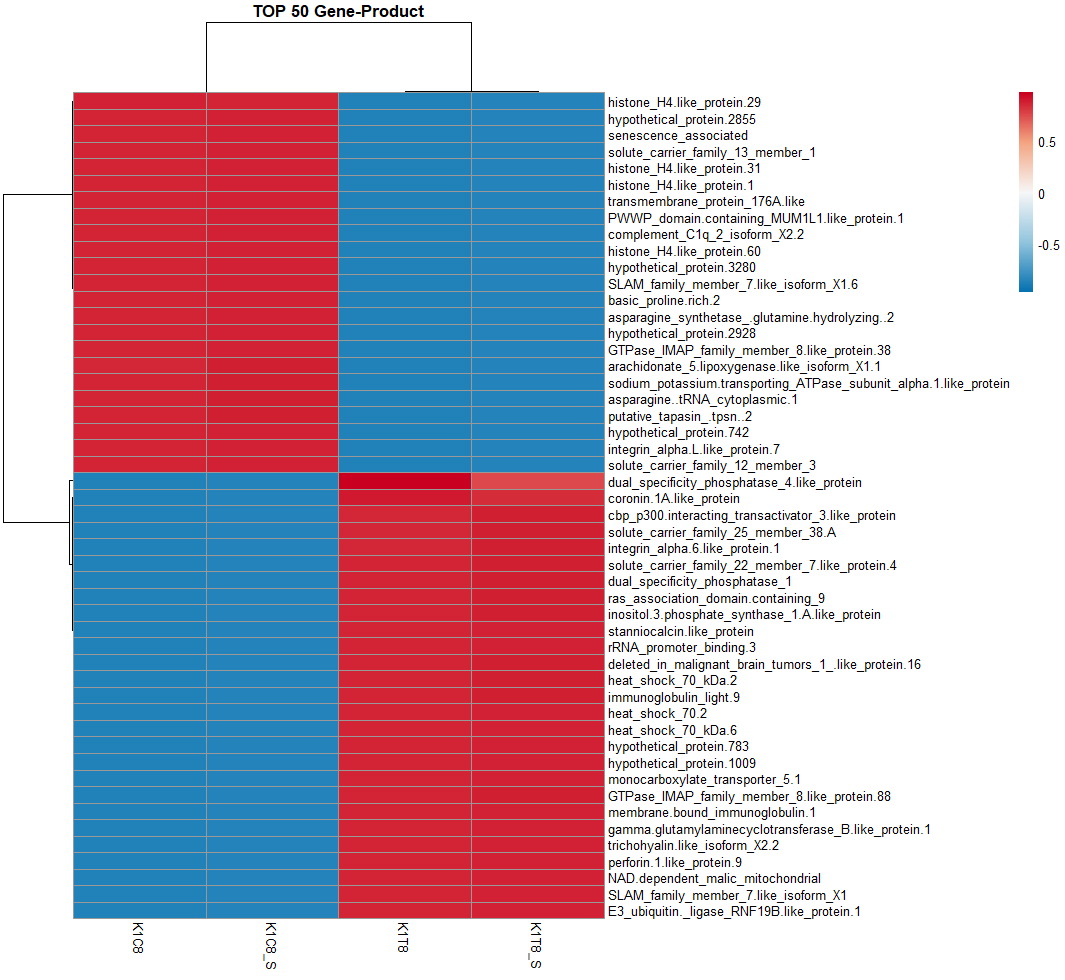
Supplementary figure 4. Heat map of RNA-seq transcriptome of *Labeo rohita* kidney for top 50 significantly differentially expressed genes between control (K1C8 and K1C8-S) and 8 ppt salinity treated samples (K1T8 and K1T8-S). The heat map showing normalized read counts (rows) for transcripts and four samples (columns). The blue and red colour corresponds to the down and up regulated genes in respective samples

**
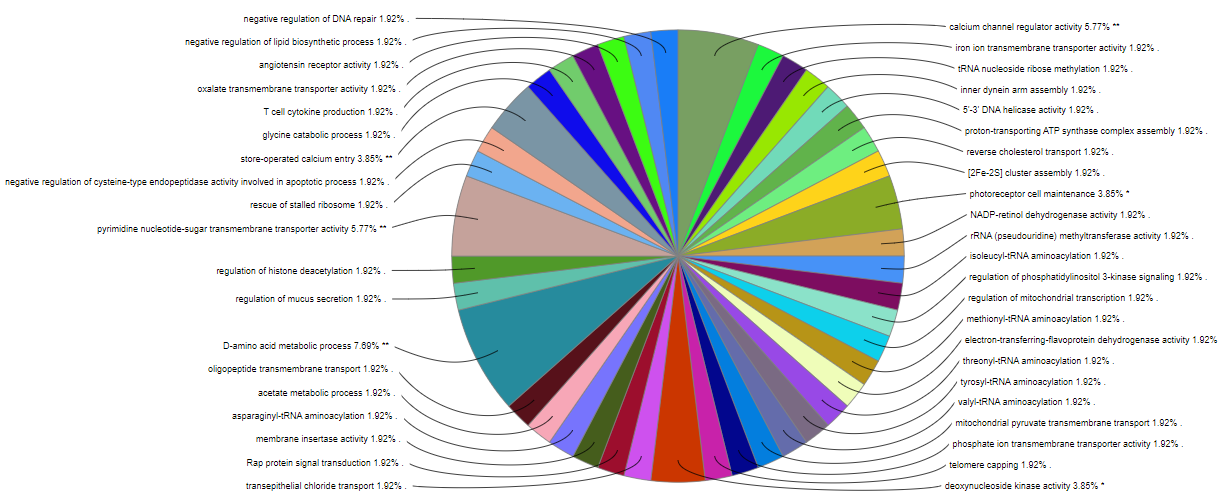
**

Supplementary figure 5. ClueGO graphical representation of percentage of differentially expressed transcripts associated with enriched GO term at 2 ppt salinity challenged *Labeo rohita* kidney transcriptome upon transferring to high salt concentration. Each colour represents individual GO term and arc length and area of each slice represents percentage of genes involved in that specific GO term.


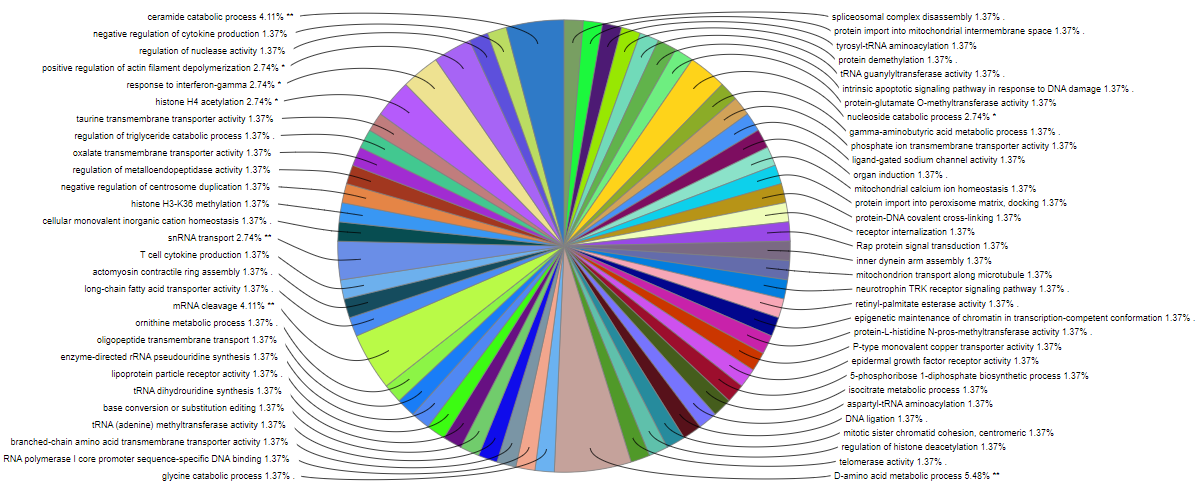


Supplementary figure 6. ClueGO graphical representation of percentage of differentially expressed transcripts associated with enriched GO term at 4 ppt salinity challenged *Labeo rohita* kidney transcriptome upon transferring to high salt concentration. Each colour represents individual GO term and arc length and area of each slice represents percentage of genes involved in that specific GO term.


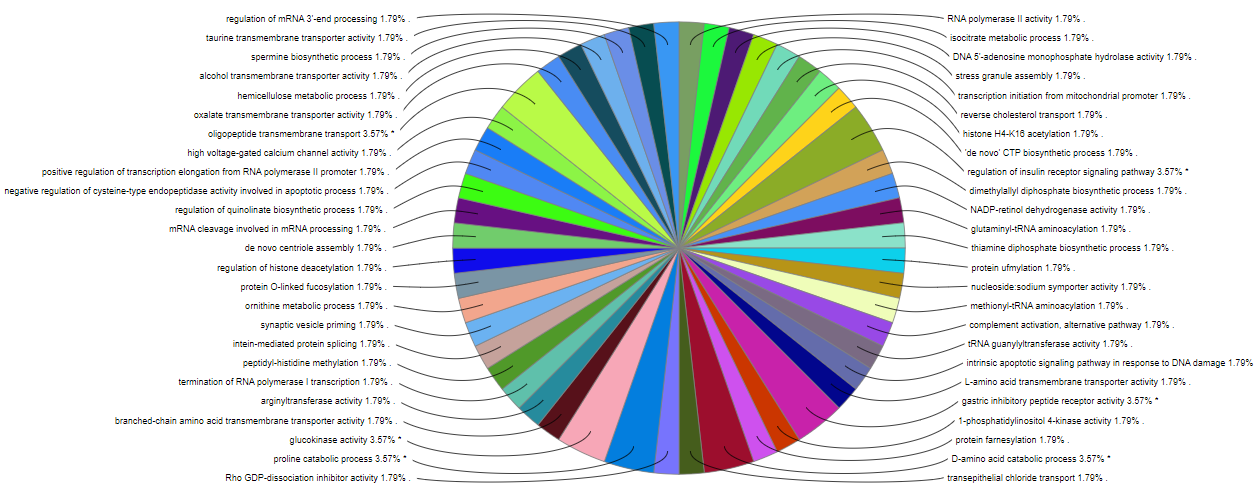


Supplementary figure 7. ClueGO graphical representation of percentage of differentially expressed transcripts associated with enriched GO term at 6 ppt salinity challenged *Labeo rohita* kidney transcriptome upon transferring to high salt concentration. Each colour represents individual GO term and arc length and area of each slice represents percentage of genes involved in that specific GO term.


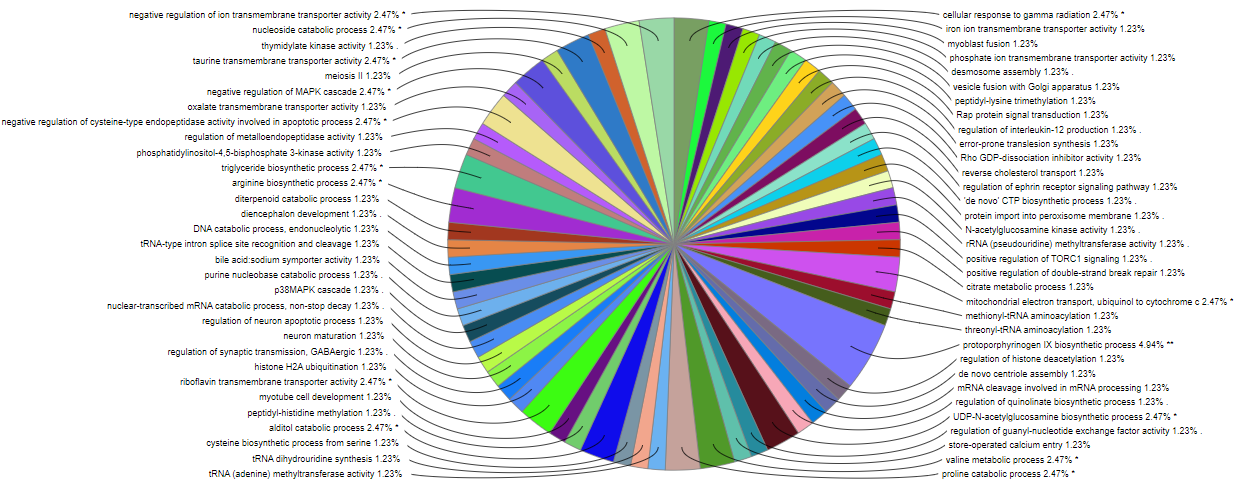


Supplementary figure 8. ClueGO graphical representation of percentage of differentially expressed transcripts associated with enriched GO term at 8 ppt salinity challenged *Labeo rohita* kidney transcriptome upon transferring to high salt concentration. Each colour represents individual GO term and arc length and area of each slice represents percentage of genes involved in that specific GO term.


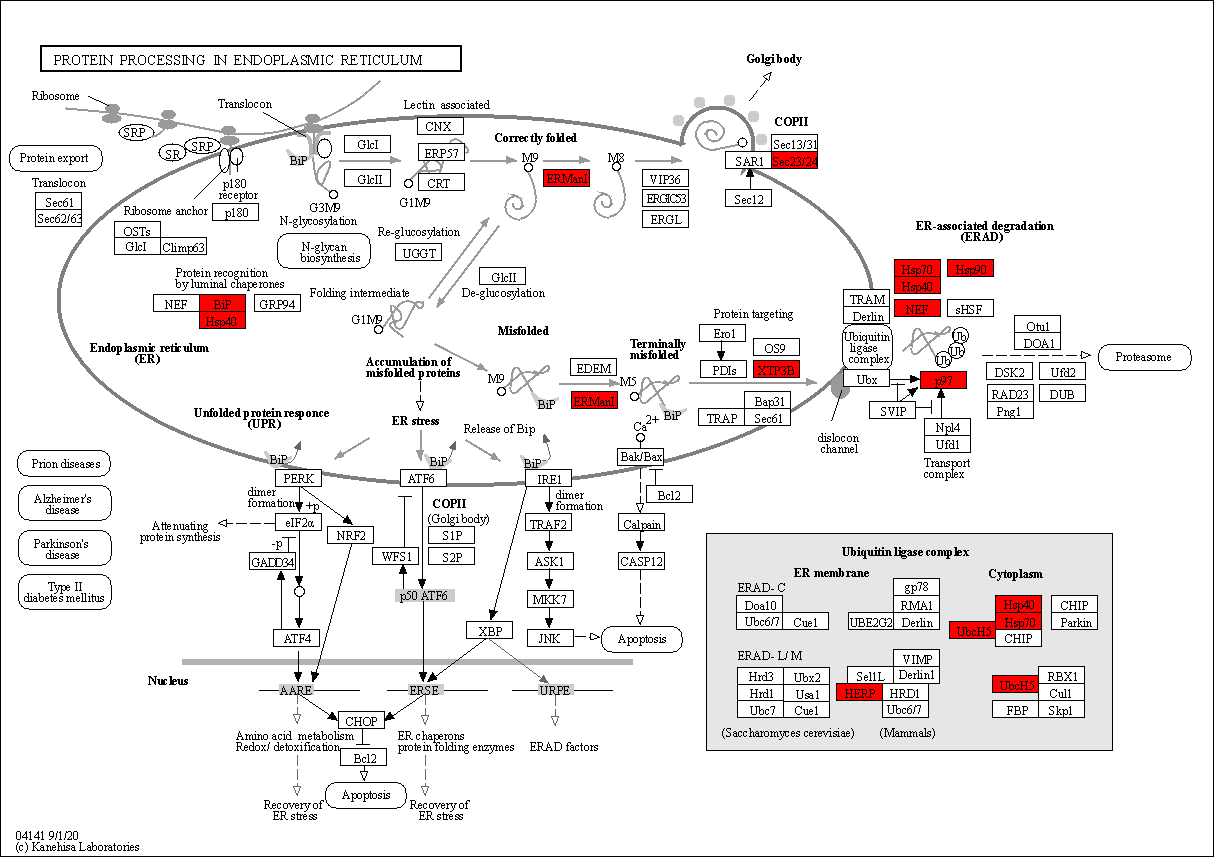
Supplementary figure 9. Differentially expressed genes involved in the endoplasmic reticulum associated degradation of terminally misfolded proteins due to hyper salinity stress of kidney tissue in *Labeo rohita* (Genes highlighted in red colour are differentially expressed in our study), BiP- Heat shock protein family 70 kDa member 5; Hsp40- DNAJ like protein 4; ERManI- mannosyl-oligosaccharide alpha-1,2-mannosidase; XTP3B- endoplasmic reticulum lectin 1; p97- transitional endoplasmic reticulum ATPase; Hsp90- heat shock HSP 90 alpha; hsp70 binding 1; HERP- homocysteine responsive endoplasmic reticulum resident ubiquitin like domain member 1; UbcH5- ubiquitin conjugating enzyme E2 D.


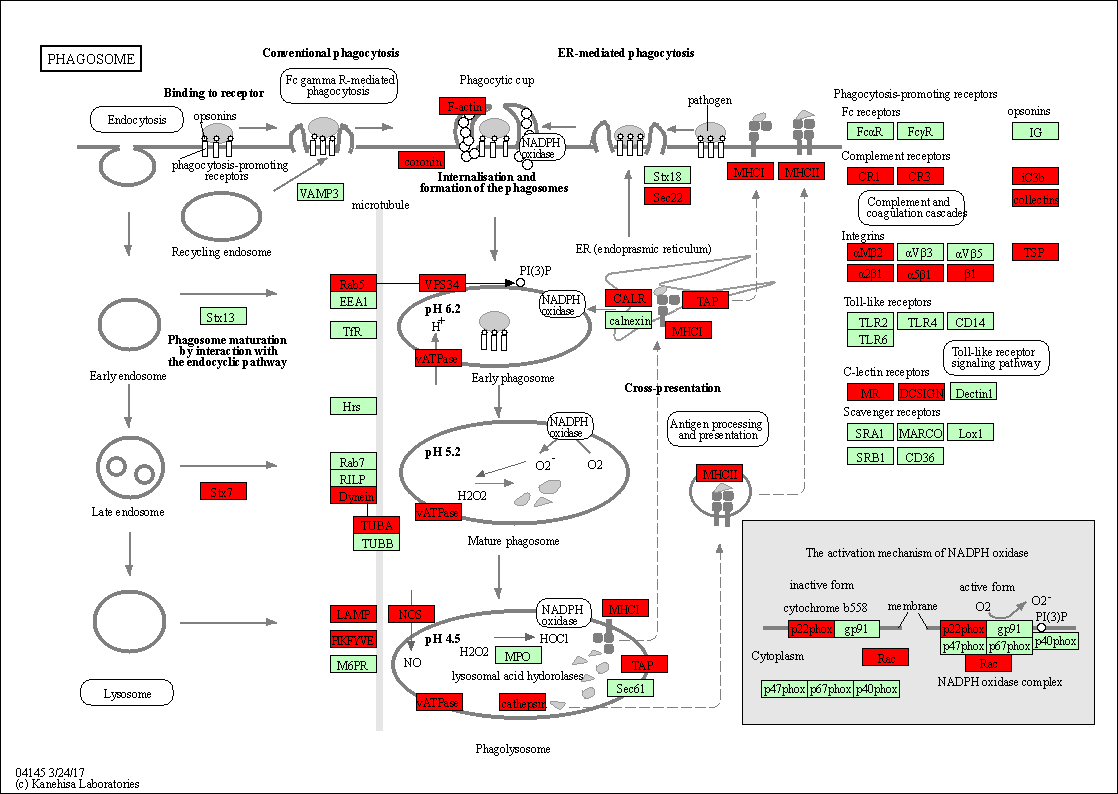


Supplementary figure 10. Phagocytic pathway enrichment to experimental salinity stress in *Labeo rohita* kidney (red highlighted genes were differentially expressed in our study). F-actin- Actin related protein complex; Coronin- Coronin 1A; Rab5-Ras related rab 5A; PIK3C3- phosphatidylinositol-3-kinase regulatory subunit gamma; ATP6V1H- V type proton ATPase subunit H; ATP6V1B2- V type proton ATPase subunit brain; Stx7-Syntaxin 7; Dynein- cytoplasmic dynein 1 heavy chain 1; TUBA- Tubulin alpha chain like protein 1; NOS- Nitric oxide synthase; Lysosomal membrane associated protein 1; PIKFYVE- zinc finger FYVE domain containing; Cathepsin- Cathepsin S; TAP- ATP binding cassette sub family B member; MHC1- Majot histocompatibility complex alpha chain; MHC II- Major histocompatibility complex II; CALR-Calreticulin; Sec22-Vescicle trafficking sec22; CR1-complement receptor type 1; CR3-integrin alpha M like protein 1; IC3b-Complement 3; Collectins-mannose binding C like protein 2; αMβ2- integrin alpha M like protein 1; α2β1-integrin beta 1 like protein 3; ITGA2 -integrin alpha like protein 2; TSP- cartilage matrix like protein; MR- macrophage mannose receptor 1; DCSIGN- C type lectin domain family 4 member M; p22phox- cytochrome b 245 heavy chain; RAC- ras related C3 botulinum toxin substrate1/2.


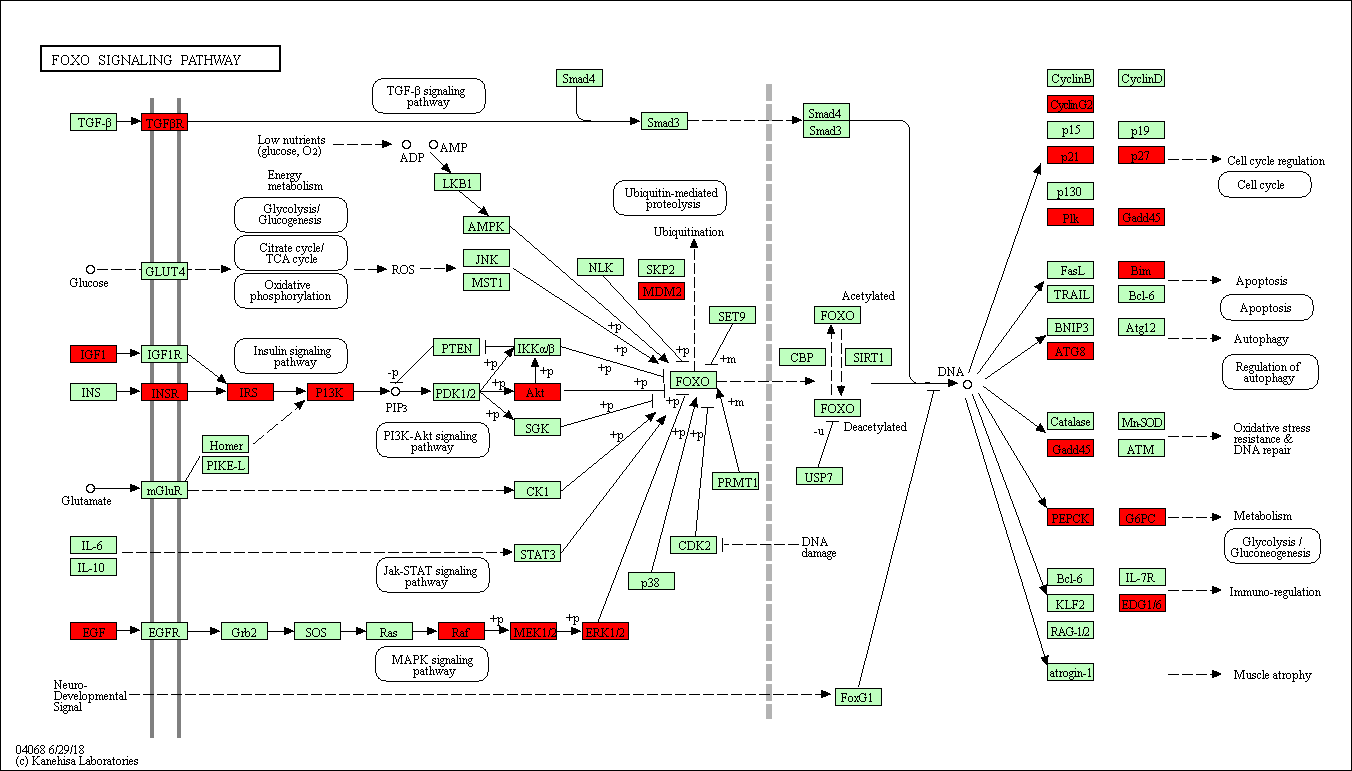
Supplementary figure 11. FOXO signaling pathway-differentially expressed genes involved under hyper salinity stress in *Labeo rohita* (red highlighted genes are differentially expressed in our study), TGFβR- TGF beta receptor type 1; IGF1-Insulin like growth factor 1; INSR- insulin receptor like protein 1, IRS- insulin receptor substrate 2; PI3K- phosphatidylinositol 3 kinase regulatory subunit; AKT1- RAC alpha serine threonine kinase; MDM2- E3 ubiquitin ligase Mdm2; EGF-epidermal growth factor; RAF1- RAF proto oncogene serine threonine kinase; MAP2K2- mitogen activated kinase kinase 2; MAPK1-Mitogen activated protein kinase 1; CyclinG2- cyclin G2 like protein; p21- cyclin dependent kinase inhibitor 1A; p27- cyclin dependent kinase inhibitor 1B; PLK- serine threonine kinase PLK3; Gadd45b- growth arrest and DNA damage inducible GADD45 beta; Bim- apoptosis facilitator Bcl 2; ATG8- gamma aminobutyric acid receptor associated like 1; PEPCK- phosphoenolpyruvate cytosolic GTP; G6PC –Glucose-6-phosphatase; EDG1/6-Sphigosine-1-phosphate receptor.


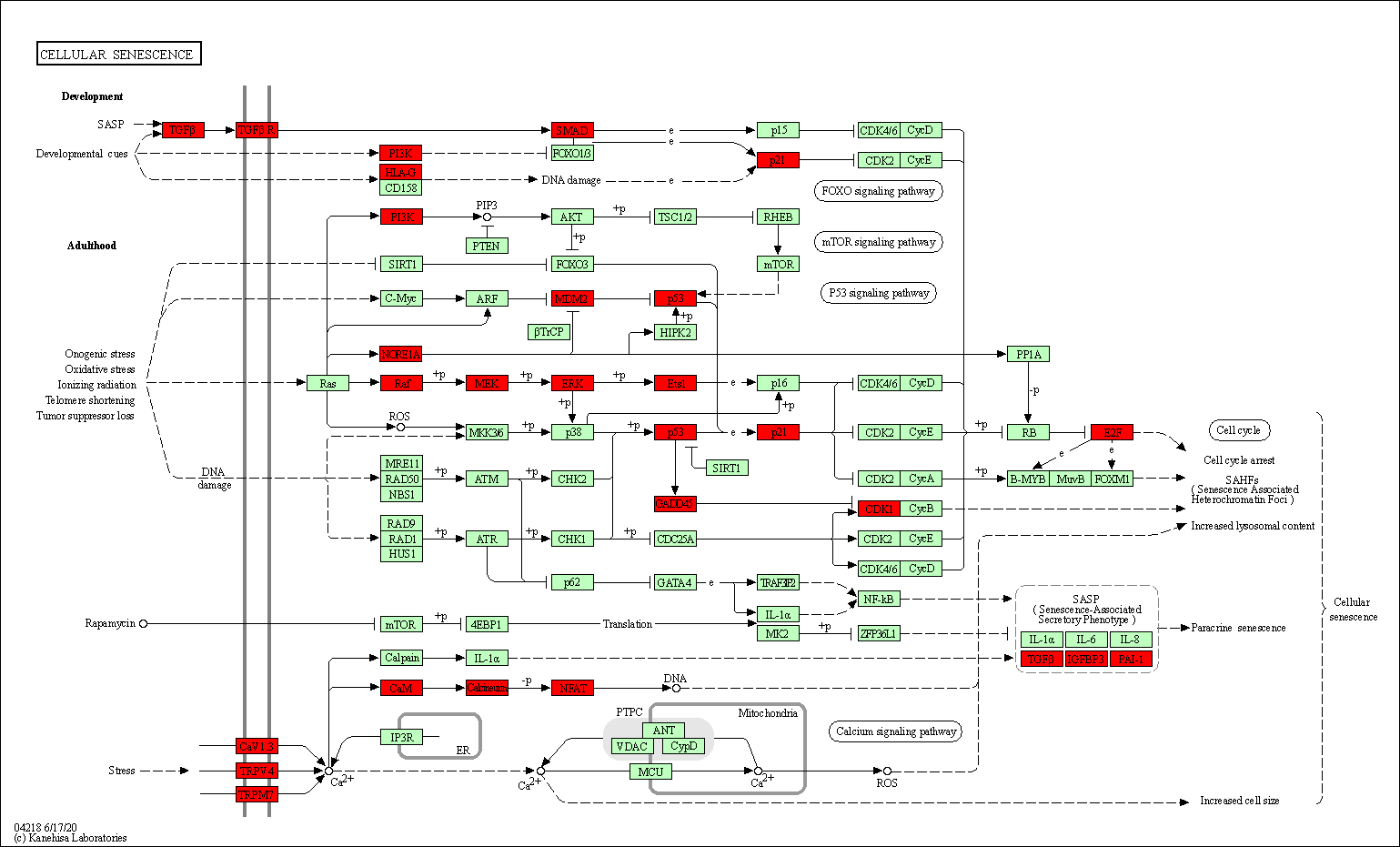
Supplementary figure 12. Differentially expressed genes involved in cellular senescence pathway of kidney transcriptome upon transfer to high salt concentration (red highlighted genes are differentially expressed in our study), TGFβ- transforming growth factor beta; TGFβR- TGF beta receptor type 1; SMAD2- mothers against decapentaplegic like protein 2; PI3K- phosphatidylinositol 3 kinase regulatory subunit; HLA-A-MHC class 1 alpha; p21- cyclin dependent kinase inhibitor 1A; MDM2- E3 ubiquitin ligase Mdm2; p53-tumor protein 53 inducible protein; NCRE1A- ras association domain containing; RAF1- RAF proto oncogene serine threonine kinase; MAP2K2- mitogen activated kinase kinase 2; MAPK1-Mitogen activated protein kinase 1; EST1- C ets 1; Gadd45b- growth arrest and DNA damage inducible GADD45 beta; CDK1- cyclin dependent kinase 1; CaV1.3- voltage dependent calcium channel subunit alpha 2 delta 1; TRPV4- Transient receptor potential cation channel subfamily V member 4; TRPM7- transient receptor potential cation channel subfamily M; CaM- Calmodulin; Calcineurin- serine threonine phosphatase PP1 gamma catalytic subunit; NFAT- nuclear factor of activated T cytoplasmic 2; E2F3/6- transcription factor E2F6 like protein; IGFBP3- insulin like growth factor binding 3; SERPINE1- putative serpin E.


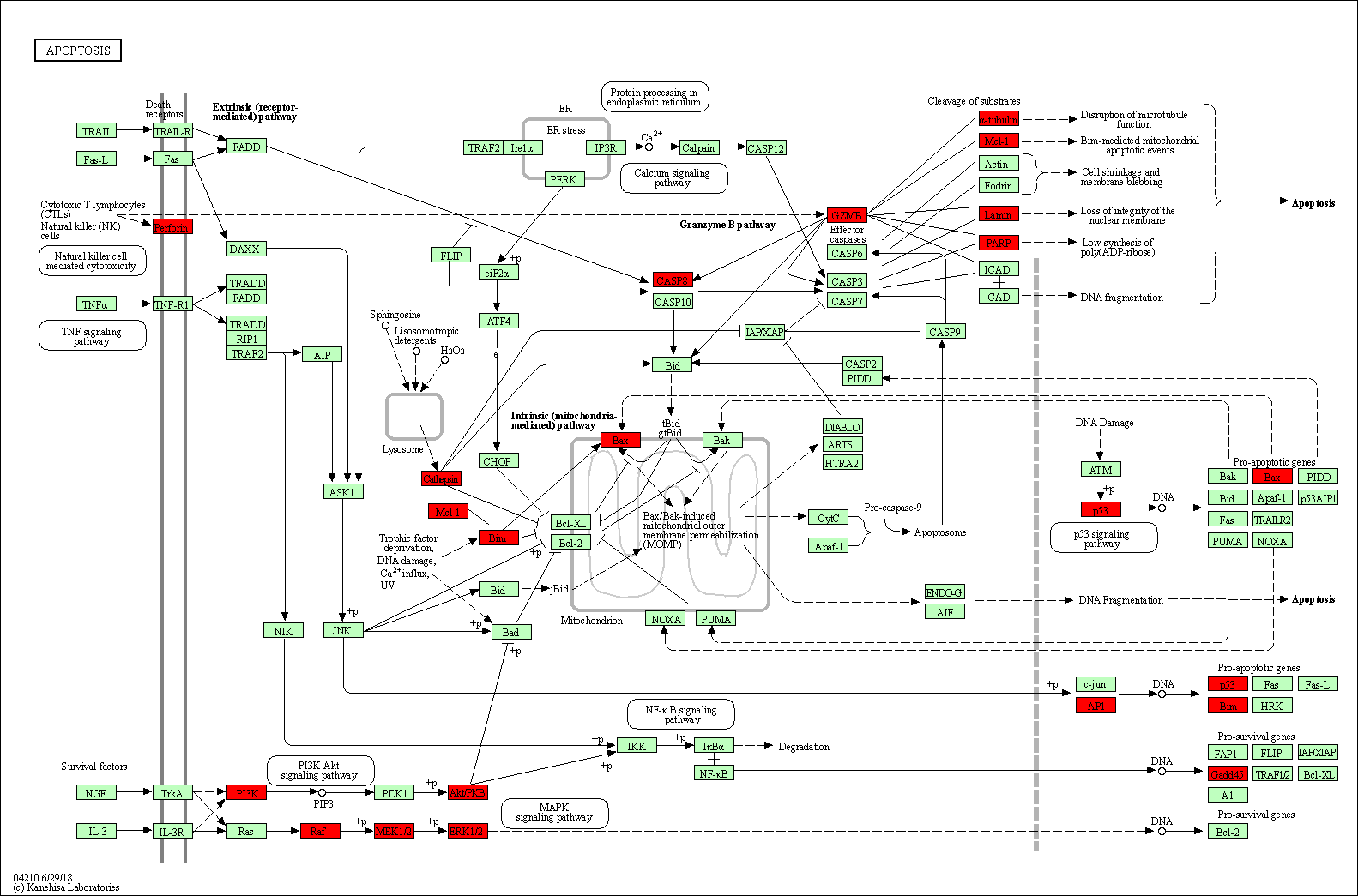
Supplementary figure 13. Differentially expressed genes of kidney transcriptome of *Labeo rohita* involved in apoptotic pathway under hypersalinity stress (red highlighted genes are differentially expressed in our study), PRF1- perforin 1 like protein; CASP8-Caspase 8; GZMB- granzyme B; α-tubulin- tubulin alpha 1B chain; Mcl-1- induced myeloid leukemia cell differentiation Mcl 1; Lamin- lamin B2; PARP- poly ADP ribose polymerase; Bax- apoptosis regulator BAX; Cathepsin –cathepsin B; Bim- apoptosis facilitator Bcl 2; p53- tumor protein 53 inducible protein; AP-1- AP 1 complex; PI3K- phosphatidylinositol 3 kinase regulatory subunit; MAP2K2- mitogen activated kinase kinase 2; MAPK1-Mitogen activated protein kinase 1; Gadd45b- growth arrest and DNA damage inducible GADD45 beta; AKT1- RAC alpha serine threonine kinase.
